# Effect of substitutional defects on resonant tunneling diodes based on armchair graphene and boron nitride nanoribbons lateral heterojunctions

**DOI:** 10.3762/bjnano.11.56

**Published:** 2020-04-24

**Authors:** Majid Sanaeepur

**Affiliations:** 1 Department of Electrical Engineering, Faculty of Engineering, Arak University, Arak, 3815688349, Iran; 2 Institute of Nanosciences and Nanotechnology, Arak University, Arak, Iran

**Keywords:** AGNR/ABNNR heterojunction, armchair boron nitride nanoribbon (ABNNR), armchair graphene nanoribbon (AGNR), negative differential resistance (NDR), nonequilibrium Green’s function (NEGF), resonant tunneling diode (RTD), substitutional defects

## Abstract

A nanometer-scaled resonant tunneling diode based on lateral heterojunctions of armchair graphene and boron nitride nanoribbons, exhibiting negative differential resistance is proposed. Low-bandgap armchair graphene nanoribbons and high-bandgap armchair boron nitride nanoribbons are used to design the well and the barrier region, respectively. The effect of all possible substitutional defects (including B_C_, N_C_, C_B_, and C_N_) at the interface of graphene and boron nitride nanoribbons on the negative differential resistance behavior of the proposed resonant tunneling diode is investigated. Transport simulations are carried out in the framework of tight-binding Hamiltonians and non-equilibrium Green’s functions. The results show that a single substitutional defect at the interface of armchair graphene and boron nitride nanoribbons can dramatically affect the negative differential resistance behavior depending on its type and location in the structure.

## Introduction

2D materials have gained tremendous research interest due to the unique properties that result from their atomic-scale thickness [1–5]. These materials, which include graphene, hexagonal boron nitride, and the large family of transition metal dichalcogenides, have electronic structures exhibiting metallic, semiconducting, and insulating properties. Novel electronic devices have been realized by heterostructures based on vertical stacking or lateral stitching of 2D materials with different electronic properties [6]. Lateral graphene/hexagonal boron nitride (Gr/hBN) heterostructures, due to very low lattice mismatch between graphene and hBN, are most suitable as platforms for fully two-dimensional nanoelectronic devices [7–11].

Resonant tunneling diodes (RTDs) are among various electronic devices realized on the platform of 2D Gr/hBN heterostructures [12–16]. RTDs exhibit negative differential resistance (NDR) and have a wide range of applications including ultra-fast switching devices, oscillators, frequency multipliers, one-transistor static memories and multi-valued memory circuits [12,17–20].

In a RTD, a material with low bandgap energy is sandwiched between two materials with larger bandgaps, i.e., a quantum well between two potential barriers, forming a so-called double-barrier quantum well structure. In the well, the energy of the electrons is quantized due to the quantum confinement of their wave function. Incident electrons with energies equal to the quantized levels of the well pass through the barriers with rather high transmission probabilities. Electrons with other energies have an extremely small chance of passing through. This causes RTDs to exhibit NDR in their current–voltage characteristic.

Conventionally, RTDs are made by vertical stacking of bulk semiconductor materials with different bandgap energies to form a planar 3D structure in which the direction of carrier transport is perpendicular to the interface of stacked materials [21–23]. In recent years, a few RTD structures based on 2D materials have been proposed [24–26]. In such RTDs the bandgap difference needed for normal operation is created by juxtaposing graphene nanoribbons (GNRs) with different widths (utilizing the inverse relation between GNR width and bandgap energy) or by periodically arranging graphene (the well) and boron nitride regions (the barriers). While the performance of conventional RTDs based on bulk semiconductors is degraded by dislocations and lattice mismatch at the interface of different bandgap materials, the RTDs based on heterojunctions between armchair graphene nanoribbons (AGNRs) and armchair boron nitride nanoribbons (ABNNRs) have shown superior performance because of the very low lattice mismatch between graphene and hBN [3]. However, inevitable interfacial defects located at the interface of Gr/hBN heterojunctions, including point defects (single vacancies and substitutional defects) and topological defects can alter the electronic properties of Gr/hBN heterostructures and, consequently, the performance of RTDs based on Gr/hBN heterojunctions [26–33]. Formation energy calculations have revealed that point defects occur preferentially at the interfaces of graphene and hBN domains rather than in the middle of these domains, and that substitutional defects are dominant [30]. Therefore, a reliable and accurate investigation of the electronic behavior in devices based on Gr/hBN heterostructures must consider the effect of such defects.

In this work, a nanometer-scaled RTD based on lateral AGNR/ABNNR heterojunctions is proposed and the effect of all possible types of substitutional defects at the interface of AGNR/ABNNR heterojunctions on the electronic behavior of the proposed RTD is investigated.

**Figure 1:**
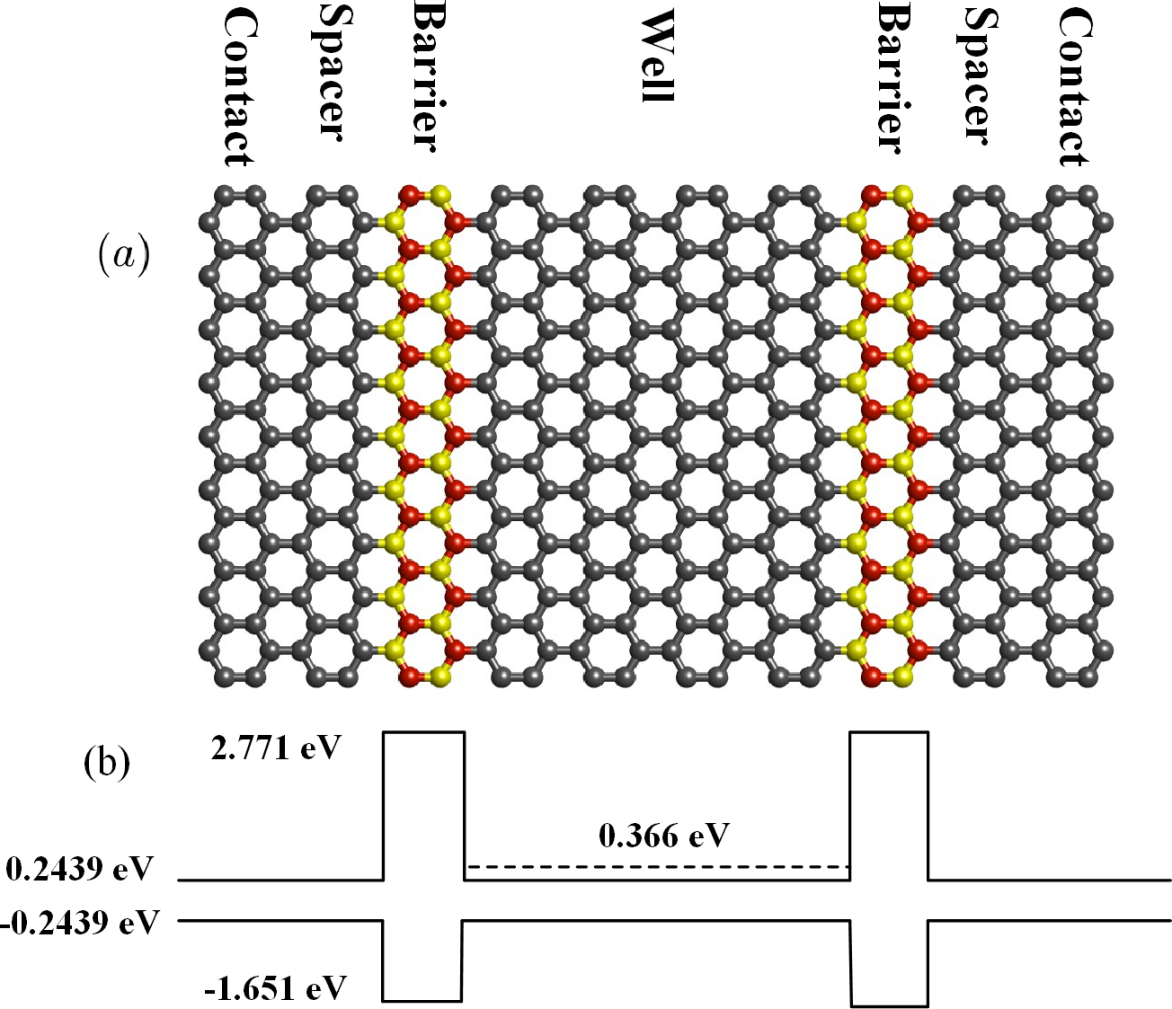
(a) Schematic representation of the proposed RTD. Carbon, boron and nitrogen atoms, are depicted in grey, red and yellow, respectively. The contacts are assumed to be heavily doped semi-infinite AGNRs. (b) Energy diagram of the proposed RTD (solid lines) and resonant energy of the well (dashed line).

The effect of substitutional defects on the electronic transport properties of zigzag graphene nanoribbons symmetrically decorated with BN is described in [34]. However, it considers the interfaces of the Gr/hBN regions are parallel to the transport direction so that there are no bandgap variations in the transport direction. Since the electron transport is in zigzag direction, spin-polarized transport calculations are utilized. Instead, in the study presented here, the electron transport is in armchair direction, which is not spin-polarized. Therefore, nonequilibrium Green’s functions with tight-binding Hamiltonians (without considering spin degree of freedom) are utilized for electronic transport calculations. Furthermore, in the proposed RTD there are four GNR/BNNR heterojunctions perpendicular to the transport direction (Figure 1), which are required to construct the double-barrier quantum well structure.

## Calculation Method

Figure 1a schematically shows the structure of the proposed RTD. An AGNR of 19 carbon atoms in width (19-AGNR) and four hexagonal carbon rings in length (1.7 × 1.6 nm^2^) is sandwiched between two ABNNRs of the same width but of only one hexagonal hBN ring in length (1.7 × 0.42 nm^2^). Two spacer regions are considered at both sides of the structure to exclude outward interfacial defects from the contact regions. The whole structure is assumed to be connected to semi-infinite AGNR contacts at both sides. The energy band edge diagram of the proposed RTD along with the quantized energy level of the well are shown in Figure 1b. Due to small lattice mismatch between graphene and hBN the edge bond relaxation correction for carbon atoms at the interface of C and hBN domains could be ignored [35]. Therefore, the electronic structure of the proposed RTD could be modeled via a tight-binding Hamiltonian, *H*_D_, including first nearest-neighbor interactions [36]. The hopping energies between C, B and N atoms and the on-site energies of B and N atoms are listed in Table 1 [37].

**Table 1:** Tight-binding parameters for the CBN composite structure^a^.

ε_B_	ε_C_	ε_N_	*t*_CC_	*t*_CB_	*t*_CN_	*t*_BN_

2.76	0.00	−1.64	2.65	2.25	1.70	2.40

^a^ε and *t* denote the on-site and hopping energies, respectively (both in eV).

In nonequilibrium Green’s functions the retarded Green’s function of the device (scattering region) is written as [38]:

[1] $$G^{r}\left(E\right)={\left[\left(E+i\eta \right)I-H_{\text{D}}-\Sigma \left(E\right)\right]}^{-1},$$

where η is an infinitesimal positive number and the non-Hermitian self-energy matrix, Σ(*E*) *=* Σ_1_(*E*) *+* Σ_2_(*E*), represents the escape rate of electrons from the device into the semi-infinite contacts. The self-energy matrices are calculated through a highly convergent recursive method [39]. Then the transmission as a function of the energy is obtained via [39]:

[2] $$T\left(E\right)=Tr\left[\Gamma_{2}\left(E\right)G^{\text{r}}\left(E\right)\Gamma_{1}\left(E\right)G^{\text{a}}\left(E\right)\right],$$

in which *G*^a^(*E*) *=* (*G*^r^(*E*))^†^ is the advanced Green's function and Γ*_j_* (*j* = 1, 2), represent the level broadening due to the coupling between device and contacts:

[3] $$\Gamma_{\text{j}}\left(E\right)=i\left[\Sigma_{\text{j}}\left(E\right)+\Sigma_{\text{j}}^{†}\left(E\right)\right],\;\;\;\;i=\sqrt{-1}.$$

Finally, the current through the device is calculated via [38]:

[4] $$I=\frac{2q}{h}\int_{-\infty }^{\infty }\text{d}E\;T\left(E\right)\left[f_{\text{1}}\left(E\right)-f_{2}\left(E-qV\right)\right]\;,$$

in which *q* is the electron charge, *f*(*E*) is the Fermi–Dirac distribution function at the contacts and *V* is the bias voltage. The Fermi level at both contacts is assumed to be 0.03 eV above the conduction band edge [40]. Room temperature (300 K) is considered in all simulations.

## Results and Discussion

**Figure 2:**
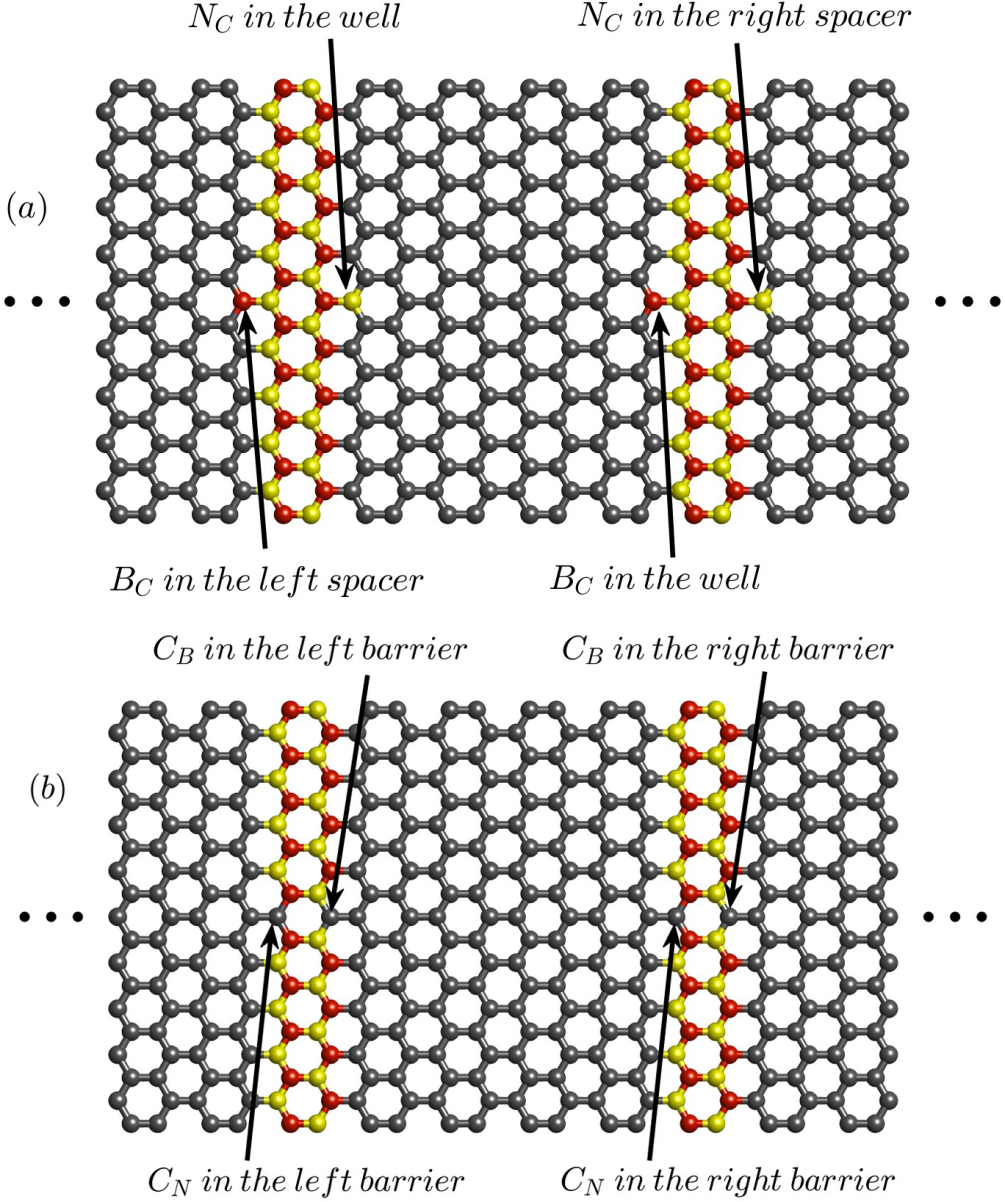
All possible substitutional defects at the interfaces of RTD based on AGNR/ABNNR heterojunctions. Carbon, boron and nitrogen atoms are depicted in grey, red and yellow, respectively.

Substitutional carbon atoms in boron or nitrogen sublattices (C_B_ and C_N_) as well as boron or nitrogen atoms on carbon sites (B_C_ and N_C_) are considered (Figure 2). Each defect could occur inside the well or in the contact regions. Since the typical defect concentration for real samples is one defect per ca. 10 Å, one defect of each type is considered at the Gr/hBN heterojunctions [41].

**Figure 3:**
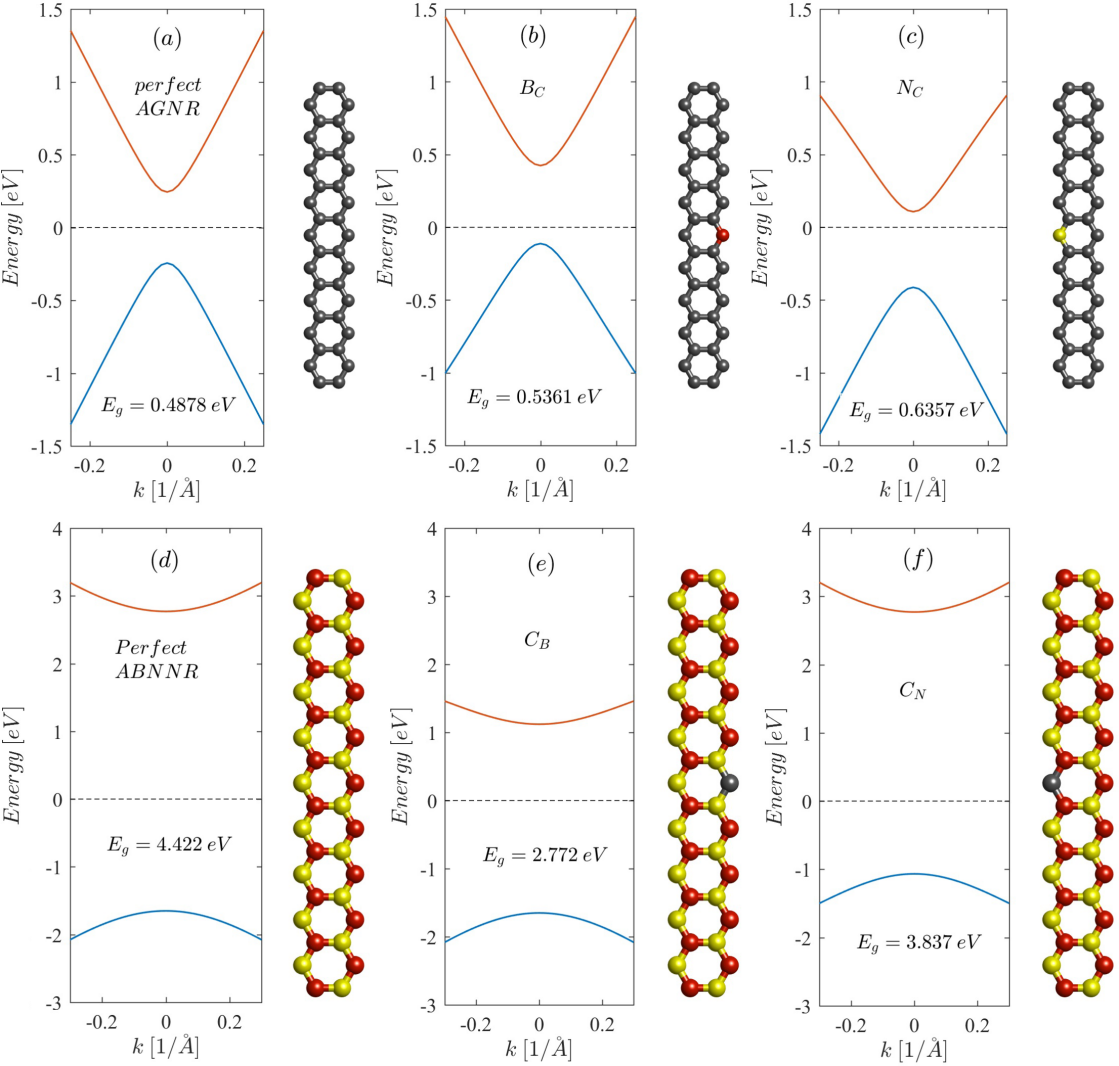
Effect of substitutional defects on the energy bandstructure of 19-AGNR and 19-ABNNR. Carbon, boron and nitrogen atoms are depicted in grey, red and yellow, respectively.

Figure 3 shows the effect of substitutional defects on the energy bandstructure of 19-AGNR and 19-ABNNR. Both B_C_ and N_C_ defects increase the bandgap of AGNR (Figure 3b,c) due to breaking the symmetry between two graphene sublattices. A B_C_ defect, due to the p-type character, shifts the conduction band edge toward higher energies, while an N_C_ defect, due to the n-type character, shifts it toward lower energies. In contrast, both C_B_ and C_N_ defects decrease the bandgap of ABNNR. However, a C_B_ defect in ABNNR, due to the n-type character, shifts the conduction band edge toward lower energies while the C_N_ defect, due to the p-type character, shifts it toward higher energies (Figure 3e,f).

**Figure 4:**
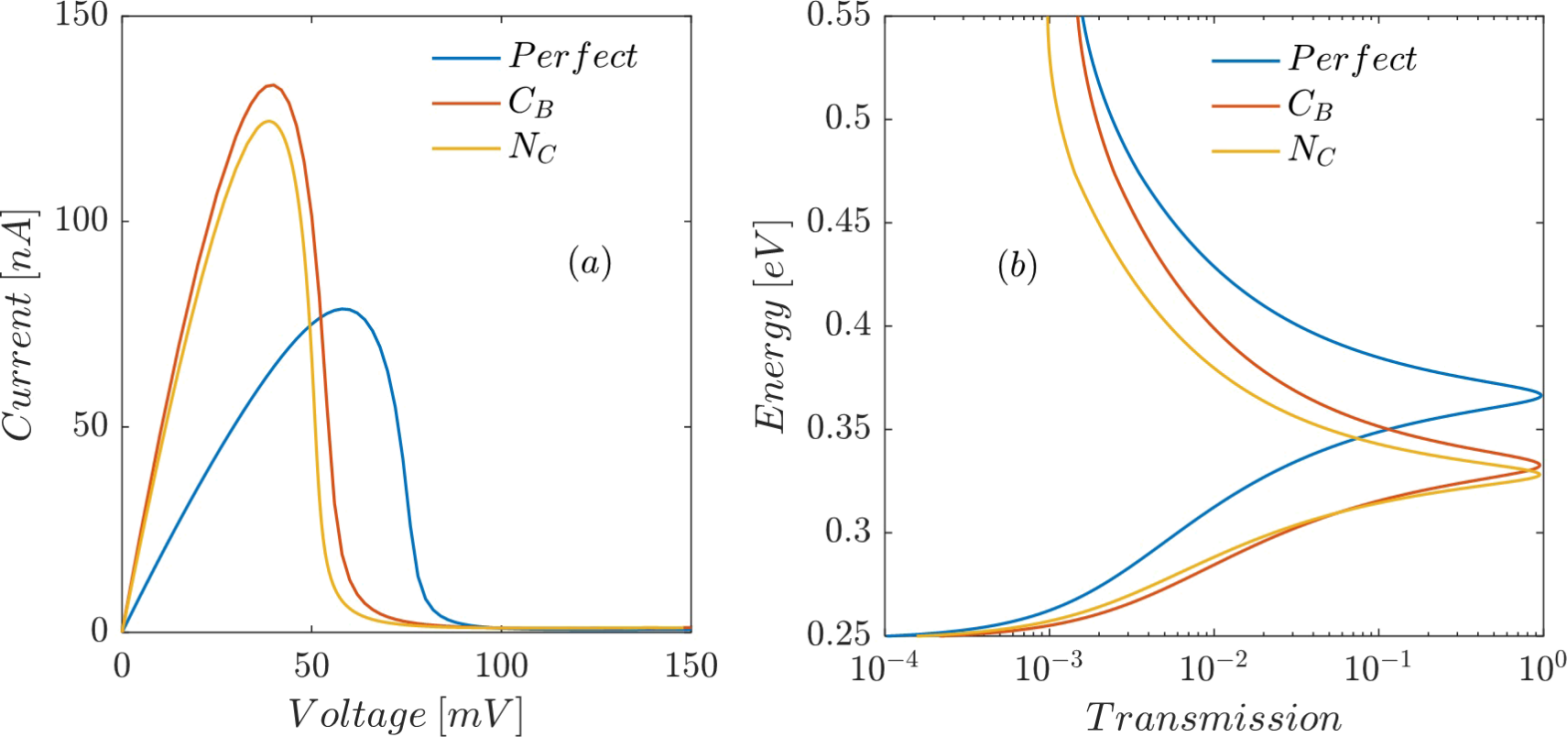
Effect of C_B_ and N_C_ defects at the interface between the left ABNNR barrier and the well on (a) the current–voltage characteristic and (b) the transmission trough the proposed RTD as a function of the electron energy.

Figure 4a depicts the effect of C_B_ and N_C_ substitutional defects at the interface between the left ABNNR barrier and the well on the current–voltage characteristic of the proposed RTD. Both defects lower the on-site energy at the place of the substituted atom due to the n-type character, which shifts all energy levels including the conduction band edge toward lower energies. The N_C_ defect inside the well also lowers the resonant energies of the well. Therefore, the transmission peaks move downward (Figure 4b), which causes the peak current (*I*_p_) to increase and the peak voltage (*V*_p_) to decrease (Figure 4a). A C_B_ defect in the left barrier lowers the conduction band edge (Figure 3e). This also, by reducing the height of the left energy barrier, lowers the resonant energies of the well, which in turn moves the transmission peaks downward (Figure 4b). Lower transmission peaks translate to decreased *V*_p_ and increased *I*_p_ (Figure 4a).

**Figure 5:**
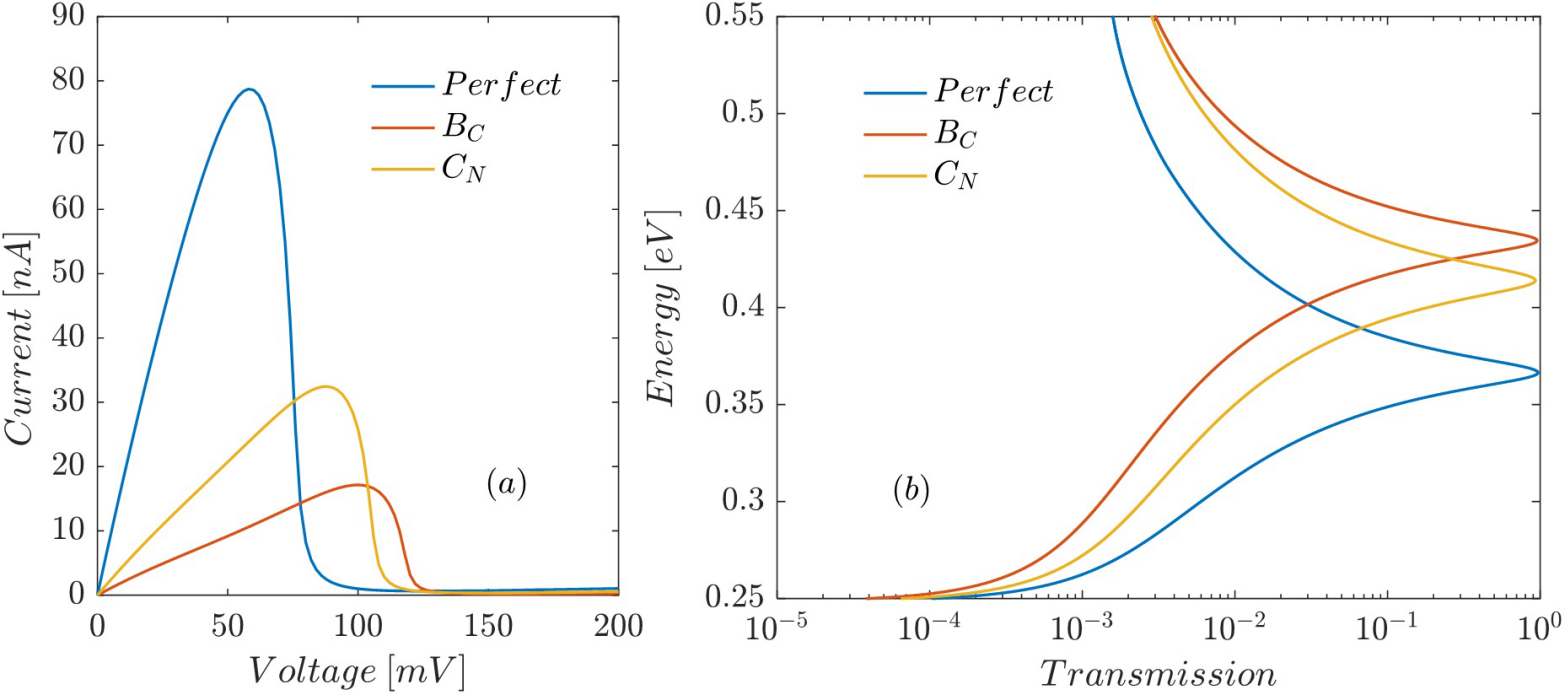
Effect of B_C_ and C_N_ defects at the interface between the right barrier and the well on (a) current–voltage characteristic and (b) the transmission trough the proposed RTD as a function of the electron energy.

Both B_C_ and C_N_ substitutional defects have p-type character (adding an extra hole to the system) because the added atom has one electron less than the removed atom. Therefore, a B_C_ defect in the well shifts all allowed energy levels (including discrete resonant energies) upward (Figure 3b), which causes *V*_p_ to increase and *I*_p_ to decrease (Figure 5a). A C_N_ defect at the interface between the right barrier and the well (inside the barrier) also moves the conduction band edge upward, which in turn shifts the resonant energies of the well upward causing *V*_p_ to increase and *I*_p_ to decrease (Figure 5a).

**Figure 6:**
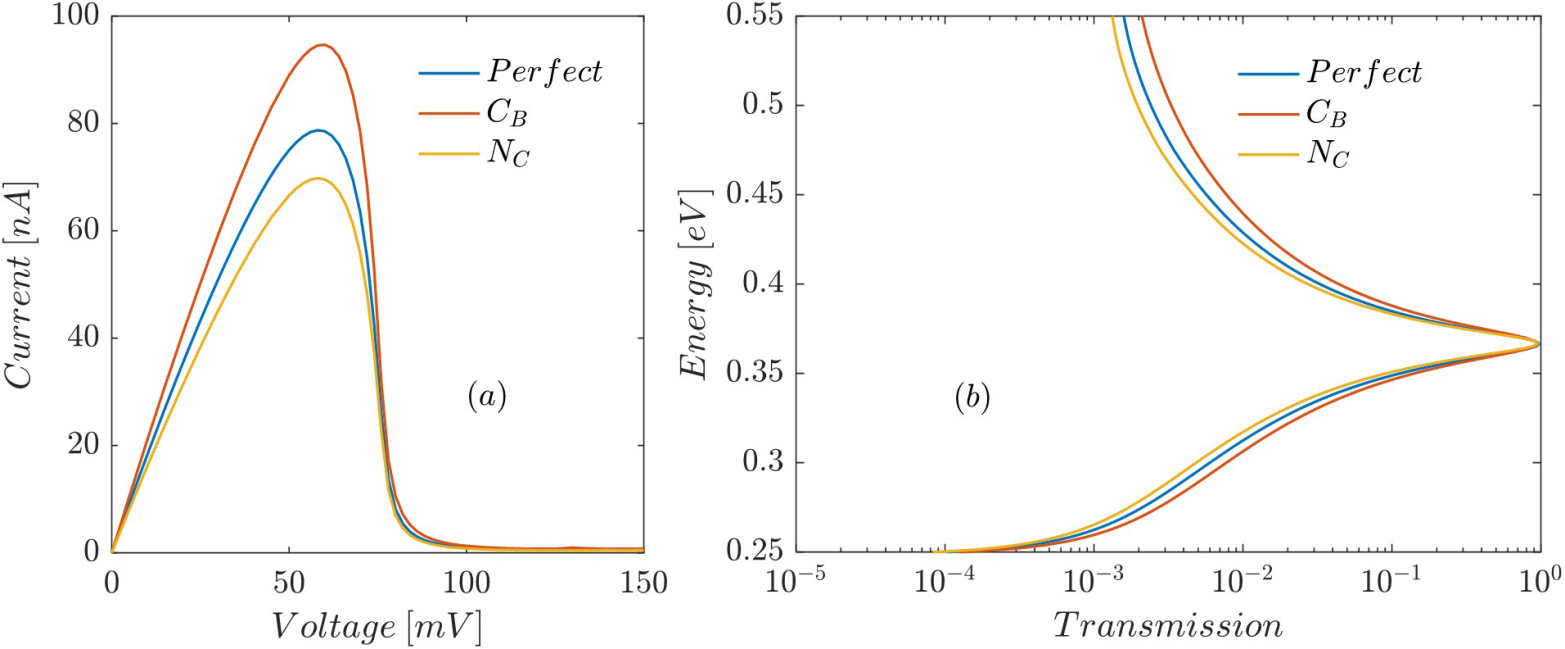
Effect of C_B_ and N_C_ defects at the interface between the right barrier and the right spacer on (a) current-voltage characteristic and (b) the transmission trough the proposed RTD as a function of the electron energy.

C_B_ and N_C_ defects may occur at the interface between the right barrier and the right spacer (see Figure 2). Because these substitutional defects are outside the well region they have no effect on the resonant energies of the well (Figure 6b). Therefore, in both cases *V*_p_ remains unchanged as illustrated in Figure 6a. Nevertheless, a C_B_ defect in the right barrier lowers the conduction band edge and consequently the barrier height. Therefore, the transmission probabilities over the right barrier at all energies are increased compared to a defect-free RTD (red curve in Figure 6b). The N_C_ defect in the right spacer also lowers the conduction band edge. This creates a local quantum well in the right spacer region, which, by localizing electron wave functions, reduces the transmission coefficients (yellow curve in Figure 6b). This causes *I*_p_ to decrease compared to a defect-free structure.

**Figure 7:**
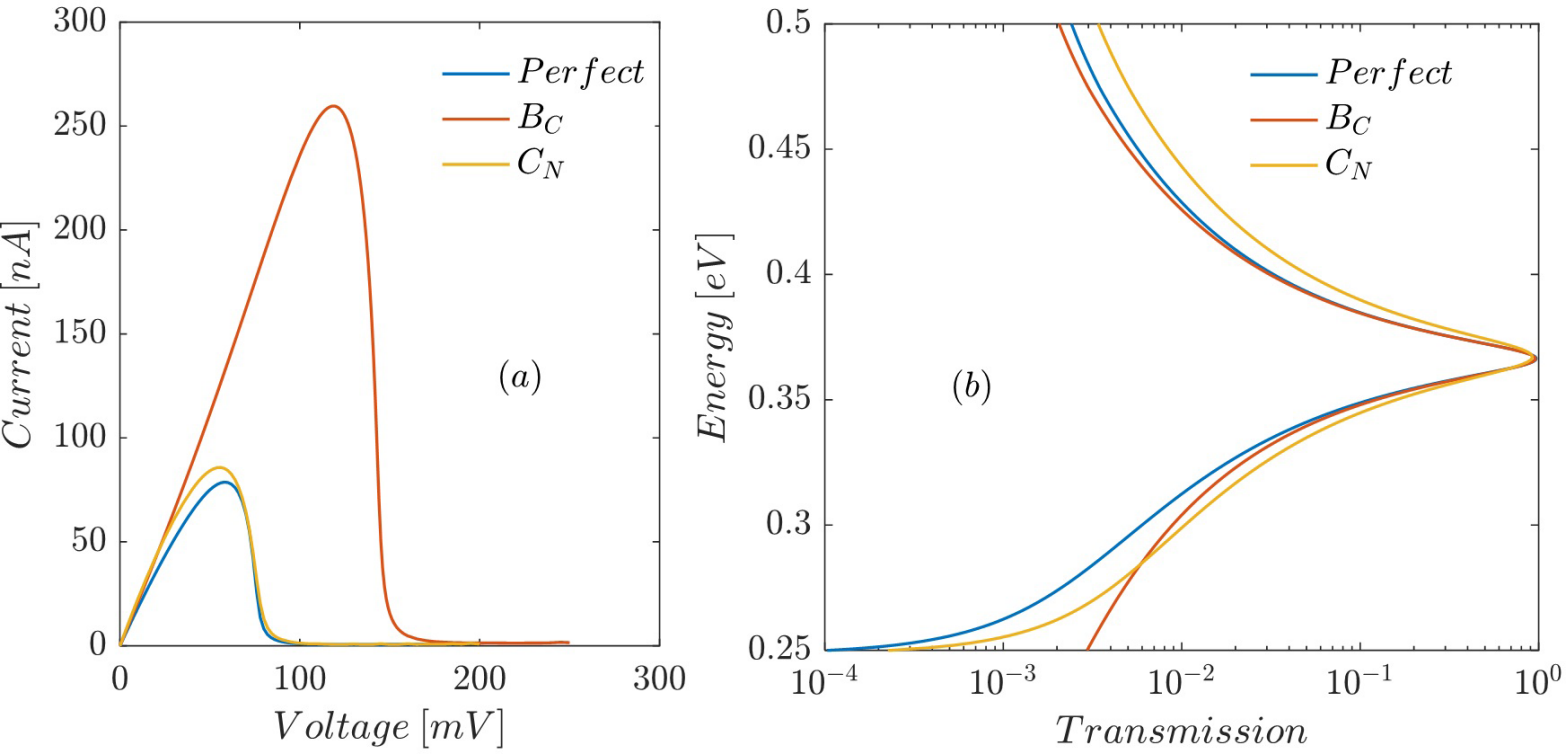
Effect of B_C_ and C_N_ defects at the interface between the left barrier and the left spacer on (a) current–voltage characteristic and (b) the transmission trough the proposed RTD as a function of the electron energy.

Figure 7 compares the current–voltage characteristic of RTDs including B_C_ and C_N_ defects at the interface between the left barrier and the left spacer with that of a defect-free RTD. If there is no defect in the left spacer, by increasing the bias voltage a quantum well is formed in this region, which localizes incident electron waves. However, a B_C_ defect in the left spacer region shifts the conduction band edge upward. Thus, a potential barrier with a very small height (with respect to the ABNNR potential barrier) is formed. Although by increasing the bias voltage the height of this potential barrier is lowered, a quantum well is never formed in the spacer region in this case (at least up to a bias voltage of 250 mV, which is considered in this work). The absence of a local quantum well in the left spacer region (for bias voltages ranging from 0 to 250 mV) causes the transmission peak to vanish at larger bias voltages with respect to the defect-free structure. Therefore, the peak values of current and voltage are increased with respect to defect-free structure (Figure 7a). A C_N_ defect in the left barrier region, besides reducing the bandgap, slightly lowers the conduction band edge (Figure 3f). This has a twofold effect: a small downward shift of the resonant energies of the well and a slight increase in transmission probabilities over the left barrier (Figure 7b), which in turn cause *I*_p_ to slightly increase and *V*_p_ to slightly decrease, as illustrated by the yellow curve in Figure 7a. Thus, a single substitutional defect can severely alter the NDR behavior of RTDs based on AGNR/ABNNR heterojunctions. This implies that by intentionally introducing such defects in devices based on AGNR/ABNNR heterojunctions, nanoelectronic devices with desired performance characteristics can be designed.

## Conclusion

The effect of substitutional defects on the NDR behavior of a nanometer-scaled RTD based on 2D heterojunctions of AGNR/ABNNR was investigated. It was shown that a single substitutional defect, depending on its type and position, could severely alter the NDR behavior of the proposed RTD. While a B_C_ defect inside the well region decreases *I*_p_ and increases *V*_p_, it increases both *I*_p_ and *V*_p_ if located at the interface between left barrier and spacer. An N_C_ defect inside the well increases *I*_p_ and decreases *V*_p_, while it decreases *I*_p_ and does not affect *V*_p_ if located at the interface between right barrier and spacer. Although a C_N_ defect inside the well has the same effect as a B_C_ defect, it increases *I*_p_ and decreases *V*_p_ if located at the interface between left barrier and spacer. A C_B_ defect inside the well has the same effect as an N_C_ defect, while it decreases *I*_p_ and does not alter *V*_p_ if located at the interface between right barrier and spacer. Substitutional defects can be intentionally incorporated in 2D heterojunctions in a controllable manner. Therefore, since peak current and peak voltage of the proposed RTD depend on the position of the resonant energy of the well, and the resonant energy itself is highly sensitive to the type and position of substitutional defects, the intentional introduction of such defects can be utilized to design nanoscale RTDs with desired NDR characteristic and RTD-based strain or pressure sensors with improved sensitivity.
